# Emerging Functions and Clinical Applications of Exosomal ncRNAs in Ovarian Cancer

**DOI:** 10.3389/fonc.2021.765458

**Published:** 2021-11-05

**Authors:** Yu Zhang, Yi-Jing Wei, Yi-Fei Zhang, Hao-Wen Liu, Yin-Feng Zhang

**Affiliations:** Institute for Translational Medicine, The Affiliated Hospital of Qingdao University, College of Medicine, Qingdao University, Qingdao, China

**Keywords:** ovarian cancer, Exosomal ncRNA, biomarker, progression, therapeutic function

## Abstract

Ovarian cancer (OC) is one of the deadliest gynecological malignancies worldwide and has a high mortality rate. Its dismal prognosis is closely related to late diagnosis and drug resistance. Exosomes are a novel means of intercellular communication that are involved in the genesis and development of tumors by delivering a variety of biologically active molecules, including proteins, lipids, and nucleic acids. As an important component, noncoding RNAs (ncRNAs) are selectively enriched in exosomes and participate in the regulation of specific aspects of OC development, such as proliferation, invasion, metastasis, angiogenesis, immune escape, and treatment resistance. Therefore, strategies that specifically target exosomal ncRNAs may be attractive therapeutic options. Exosomes are readily available in almost all types of human biological fluids and are biocompatible, making them promising biomarkers of OC as well as targets for therapeutic applications. In this review, we briefly summarize the biology of exosomes, the function of exosomal ncRNAs in OC development, and their potential clinical applications as biomarkers and therapeutic tools. Ideally, exosomal ncRNAs will become increasingly valuable in the diagnosis and treatment of OC in the near future.

## Introduction

### Ovarian Cancer

Ovarian cancer (OC) is one of the deadliest gynecological malignancies in the world (1). It is the seventh most common cancer and the fifth leading cause of cancer-related death (2). The etiology of OC is not well known, but multiple genetic and environmental factors have been identified as potential contributors to its pathogenesis (3, 4).

OC is distinguished according to the biological behavior of the tumor and related risk factors and can be divided into three categories: stromal, germ and epithelial cell tumors (5). Epithelial tumors are the most common subtype, accounting for 90% of all cases, and can be classified as type I and type II tumors (6). Type I cancers, including clear cell, mucinous, endometrial, and low-grade serous cancers, are usually diagnosed early in the disease, while type II cancers, including high-grade serous cancers, are often diagnosed late in the disease, and are associated with low survival (6, 7).

The majority of OC is not diagnosed at an early stage, which directly leads to its high mortality rate (8). Patients with early-stage OC often have vague symptoms, including nonspecific conditions such as pelvic pain, abdominal pain, early satiety, and abdominal enlargement, and are often misdiagnosed to as other nongynecological diseases (9). As a result, there are no specific indicators until the disease has spread to the entire abdomen, and 75% of cases are usually diagnosed at an advanced stage (10). Although pelvic examination, transvaginal ultrasound, and serum carbohydrate antigen 125 (CA125) are routine diagnostic methods for OC, their diagnostic value is limited due to low sensitivity and specificity (11). For example, CA125 is elevated in only 50% to 60% of patients with stage I or II OC (12).

At the same time, chemotherapy resistance also contributes to the high mortality of OC to a large extent (13). Acquired resistance to chemotherapy is an ongoing challenge in the treatment of patients with ovarian cancer (6). The current standard of chemotherapy for women with ovarian cancer is platinum and paclitaxel (14). Patients initially respond well to these treatments, but most relapse within 18 months, usually due to chemotherapy resistance (15). This resistance becomes apparent when patients are given chemotherapy again after their disease has relapsed (6). The 10-year survival rate is only 10–15% (16).

Taken together, it is imperative to develop new methods to effectively detect OC in its early, curable stage, and efforts are needed to combat its resistance.

### The Biogenesis and Characteristics of Exosomes

Exosomes were originally thought to be the product of membrane shedding (17). They were first described and named “exosomes” by Johnstone in 1987 (18). The biogenesis of exosomes begins with the formation of endosomes by plasma membrane endocytosis, which mature into multivesicular endosomes (MVEs). This is followed by the emergence of exosomes in the endosome system as intracavitary vesicles (ILVs), which are formed by the inward budding of the limiting membrane of MVEs (19) and may contain proteins, lipids, ncRNAs, and various other molecules (20). In general, MVEs either fuse with lysosomes for degradation or with the plasma membrane to secrete ILVs (i.e., exosomes) into the extracellular space (19). The mechanism of exosome biogenesis involves several factors, of which the most widely known regulator is endosome sorting complex required for transport (ESCRT) (21). The ESCRT system comprises ESCRT-0, -I, -II, and -III, which act in a stepwise manner wherein ESCRT-0 and ESCRT- I cluster specific substances and concentrate them in the endosomal membrane, the ESCRT-II complex is involved in the budding of the endosomal membrane, and ESCRT-III is recruited for ILV cleavage (22).

**Figure 1:**
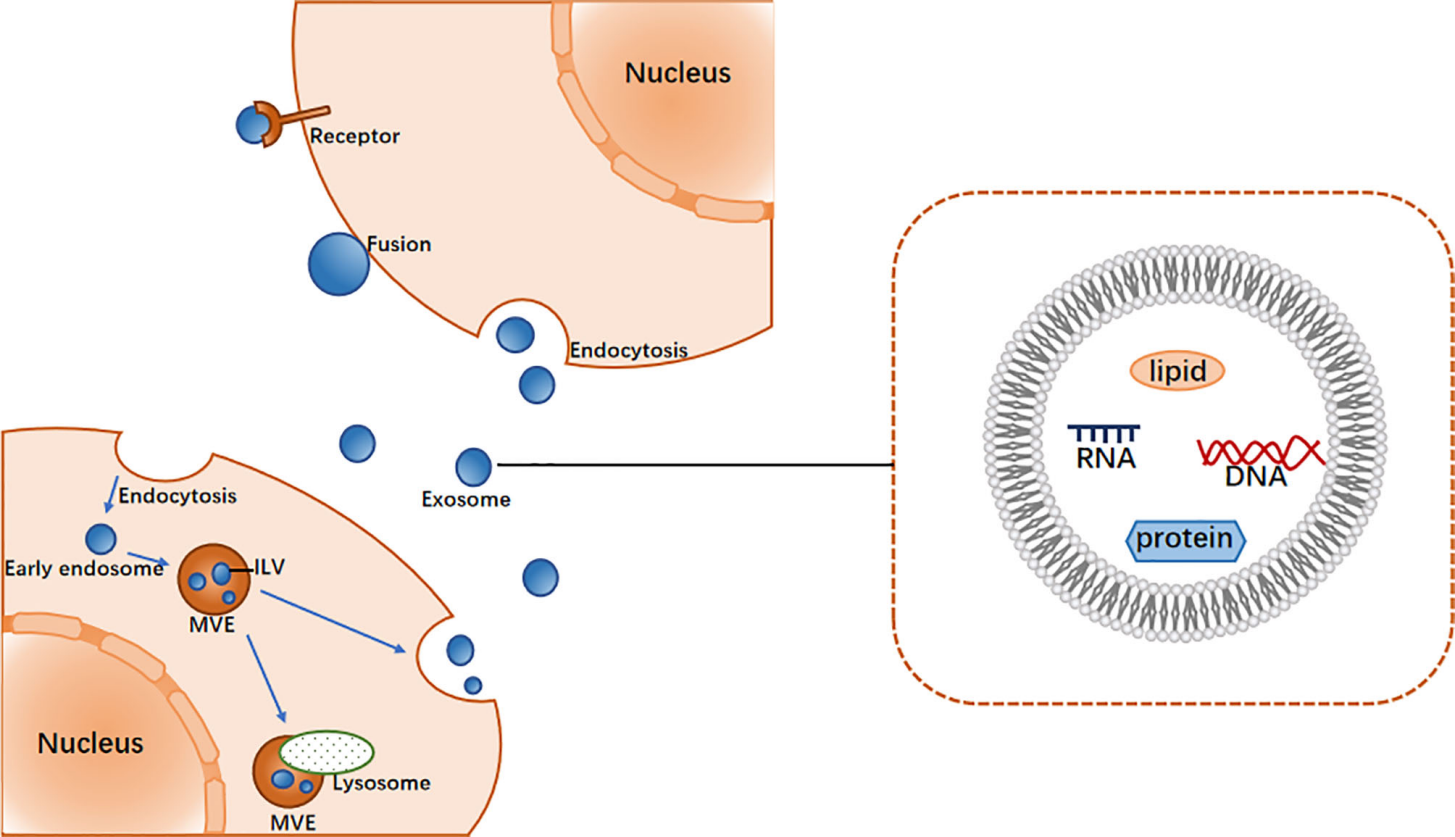
The main process of exosome biogenesis and release.

After exosomes are secreted from cells, they interact with neighboring or distant recipient cells *via* ligand/receptor interactions, direct membrane fusion and endocytosis, the result of which is the internalization of substances into the cytoplasm of the recipient cells, thereby regulating the activity of the recipient cells (**Figure 1**) (23).

The release of exosomes into the extracellular environment was originally considered a means of eliminating unwanted intracellular substances, and its biological significance has long been neglected (19). However, extensive studies have shown that exosomes are key mediators of intercellular communication (6, 17), and different exosomes may have different biological activities. Their composition depends on cell origin and is highly variable (24). It has been gradually recognized that exosomes can be involved in the pathogenesis of many diseases, including cancer (25–27). Tumor-derived exosomes can change the behavior of surrounding stromal cells and ultimately create an appropriate microenvironment for tumor growth (28). Thus, tumor and stromal cells contribute to tumor proliferation (29), invasion (30), angiogenesis (31), immune escape (32), metastasis (31), and therapeutic resistance (33) through a complex interaction of exosomes.

In addition, exosomes are widely found in a variety of biological fluids, such as blood, urine, and saliva (34). The concentration of exosomes in cancer patients is higher than that in healthy individuals, and the substances in exosomes can reflect the origin cells and real-time disease status (17). Therefore, exosomes are being investigated as potential biomarkers for the diagnosis, prognosis, and treatment of cancer (35). More importantly, because exosomes are stable and have low immunogenicity, they have also been developed as carriers to carry drugs and antitumor nucleic acids to treat cancer (36).

Although the study exosomes is relatively new, they have been involved in OC research and are expected to have applications in OC.

### Noncoding RNAs in Exosomes

As a new means of communication between cells (19), exosomes have attracted much attention in recent years because they can carry a variety of bioactive molecules regulating the activity of receptor cells, including proteins, lipids, and nucleic acids (23). These molecules are significantly protected from proteases, nucleases, and other environmental influences by the lipid bilayer membrane of exosomes (37). In addition, these molecules in exosomes are selectively packaged, secreted, and transferred between cells (6) and are highly variable according to the parental cell and pathophysiological conditions (38).

Among these bioactive components, noncoding RNAs (ncRNAs) are enriched and stable in exosomes and include microRNAs (miRNAs), long noncoding RNAs (lncRNAs), circular RNAs (circRNAs), piwi-interacting RNAs (piRNAs) and tRNA-derived small noncoding RNAs (tsRNAs) (39), all of which play important roles in a variety of pathophysiological processes (40, 41), especially in cancer (42). Interestingly, ncRNAs are selectively enriched in exosomes, and exosomal ncRNAs play biological roles in recipient cells, affecting key processes in tumor development such as oncogenesis, tumor metastasis, angiogenesis, immune regulation, and drug resistance (26, 30, 43).

In addition, due to their abnormal expression, exosomal ncRNAs are a promising source of biomarkers and potential therapeutic targets (6, 39, 44). In this review, we mainly focus on the biological functions and their emerging contributions in the diagnosis and treatment of OC.

## Exosomal ncRNAs and OC Progression

### Exosomal ncRNAs and OC Growth

As malignancies of the female reproductive system, OC is characterized as having rapid proliferation and extensive invasion, which are the most critical growth characteristics and may eventually lead to incomplete surgical resection and inevitable tumor recurrence (45). Therefore, the identification of relevant key molecules is crucial to their therapeutic efficacy. A growing body of evidence suggests that exosomal ncRNAs play important roles in the progression of OC.

**Figure 2:**
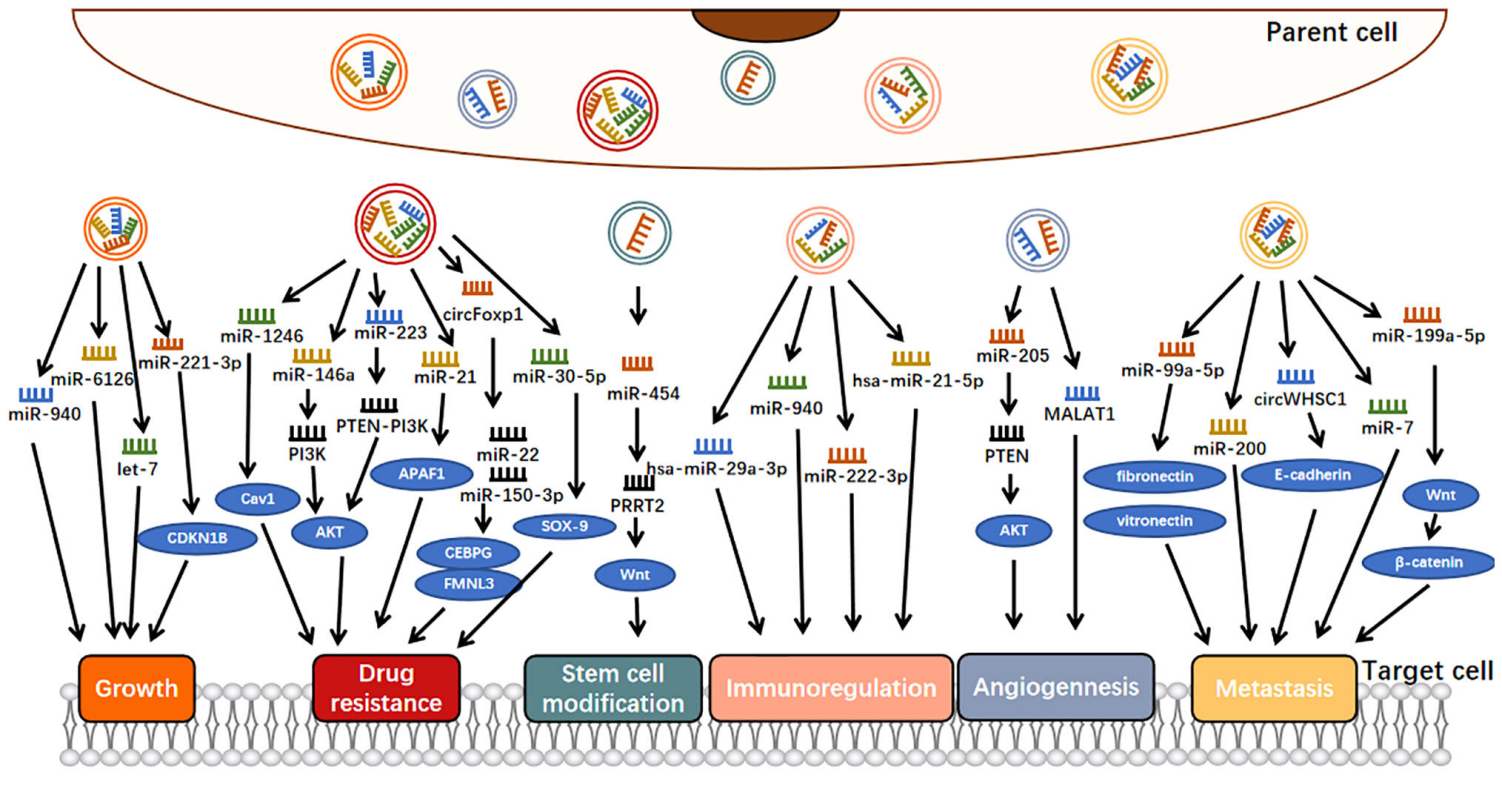
Molecular mechanisms of exosomal ncRNAs involvement in ovarian cancer progression.

Exosomes can engage in cell-to-cell communication, jettison tumor suppressor ncRNAs, and thus maintain an endogenous balance between tumor suppressor ncRNAs and their oncogenic targets, ensuring normal tumor growth (29). For example, overexpression of miR-940 could inhibit the proliferation and colony formation of OC cells and induce G0/G1 cell cycle arrest so that OC cells would secrete miR-940 into the extracellular environment *via* exosomes to maintain their proliferation and invasion (**Figure 2**) (46). Also involved in tumor cell proliferation is exosomal miR-221-3p, which is enriched in M2 exosome and can directly inhibit CDKN1B and participate in G1/S transition of EOC cells, thus playing a role in regulating EOC progression (47). Similarly, miR-6126, a tumor suppressor that targets integrin-β1 *via* exosomes, was widely applied to chemotherapy-sensitive and chemotherapy-resistant OC cells *via* exosomes, where the expression of miR-6126 was significantly higher than that of its source cells to maintain its growth process (29). In addition, let-7 could inhibit cell proliferation as a tumor suppressor, and it has been shown that let-7 levels in exosomes derived from highly invasive OC were higher than those in exosomes derived from less invasive OC, suggesting that cancer cells might promote their oncogenic properties by suppressing the expression of the let-7 family (30).

These facts are consistent with the hypothesis that malignant cells release their tumor suppressor ncRNAs *via* exosomes into the extracellular environment to maintain and promote tumorigenesis at the intracellular level (29).

### Exosomal ncRNAs and OC Metastasis

Metastasis, the leading cause of tumor-related death, is the end product of a multistep cellular biological process known as the invasion-metastasis cascade (48). Different from most other cancers, OC generally does not metastasize through the blood but rather *via* direct extension or detachment from the primary tumor to the peritoneum, passive transport *via* peritoneal fluid or ascites, where the cells spread in the peritoneal cavity and often adhere and metastasize to the greater omentum (49). Many studies have shown that exosomal ncRNAs are involved in the migration of OC.

Some OC-associated exosomal ncRNAs can be secreted by OC cells to promote the metastasis of OC (50). For example, exosomal miR-99a-5p from EOC cells affected human papilloma cells by upregulating fibronectin and vitronectin, thereby promoting cell metastasis (**Figure 2**) (51). Many studies have shown that both fibronectin and vitronectin play important roles in the peritoneal spread of OC (52, 53). Similarly, another study showed that miR-200 could only be detected in OVCAR-3 exosomes because miR-200 transcripts were expressed in OVCAR-3 cells and lost in the more aggressive SKOV-3 cell lines (30). As miR-200 regulates epithelial-mesenchymal transformation (EMT), these findings suggest that the miR200 family participates in tumor suppression by inhibiting epithelial-mesenchymal transformation, the initial step of metastasis (54). The expression of miR-200s in OVCAR-3 cells may be an important factor in determining their low invasive potential. Some exosomes secreted by OC cells are also involved in the inhibition of OC metastasis. For example, the exosomal miR-199a-5p secreted by hypoxia OC cells plays a negative regulatory role in tumor metastasis, which is achieved through HIF-2α family regulating the Wnt/β-catenin pathway (55).

In addition to the literature on miRNAs, studies have shown that circRNAs are also involved in the metastasis process of OC. For example, exosomal circWHSC1 could be transferred to peritoneal mesothelial cells, and the expression of MUC1 in mesothelial cells was upregulated and transformed them into malignant mesothelial cells, which was conducive to peritoneal spread and tumor implantation (50). In addition, E-cadherin expression was downregulated in HMRSV5 cells after incubation with exosomal circWHSC1 (50). E-cadherin is one of the key molecules involved in epithelial cell adhesion; thus, OC cells with low expression of E-cadherin were more aggressive (56).

As a medium of interaction between OC and macrophages, exosomal ncRNAs can come not only from OC cells but also from macrophages (49, 57). Related studies have shown that under stimulation of the tumor necrosis factor-like weak inducer of apoptosis (TWEAK), a member of the TNF superfamily (58), the level of miR-7 in the exosomes secreted by macrophages increases, and after these exosomes are internalized by EOC cells, the metastasis of EOC cells is inhibited by blocking the EGFR/AKT/ERK1/2 pathway (57). This study suggests that exosomal miR-7 can be a promising therapeutic target for OC *in vivo*.

In addition, ascites-derived exosomes (ADEs) play an important role in the development of ovarian cancer. The analysis suggested that miR-6780b-5p may be a key miRNA promoting tumor metastasis in ADE. ADEs transferred miR-6780b-5p to OC cells, promoting EMT and ultimately promoting OC metastasis (59).

### Exosomal ncRNAs and OC Angiogenesis

Angiogenesis has been found to be a prerequisite for tumor metastasis (60). Active angiogenesis provides adequate nutrients and oxygen to tumor cells (61). Due to hypoxia within tumors, tumor cells can release soluble factors, such as vascular endothelial growth factor (VEGF) and exosomes, which promote pathological angiogenesis (62). Folkman showed that tumors depend on the constant growth of new blood vessels and that interrupting the blood supply to tumors should eliminate cancer (61). In recent decades, many angiogenesis inhibitors have been recommended for treatment and approved for many cancers (63). However, the efficacy of many angiogenesis inhibitors is not satisfactory, partly due to the incomplete understanding and application of the molecular mechanisms of tumor angiogenesis (63).

Here, we investigated the role of exosomal ncRNAs as potential angiogenic factors in OC metastasis (**Figure 2**). A study by He revealed that serum exosomal miR-205 induced angiogenesis through the PTEN-AKT pathway, the expression of miR-205 was significantly upregulated in patients with metastatic OC compared with patients with nonmetastatic OC, and this upregulation was positively correlated with high microvascular density (31). Metastatic-associated lung adenocarcinoma transcript 1 (MALAT1) is a well-known lncRNA associated with cancer angiogenesis (60). Studies have shown that MALAT1 is increased in exosomes secreted by EOC cells, can be transferred to recipient human umbilical vein endothelial cells (HUVECs), affects HUVECs by stimulating the expression of angiogenesis-related genes, and ultimately promotes angiogenesis and tumor metastasis (26). These findings demonstrated an exosome-dependent mechanism of tumor angiogenesis, suggesting that exosomal ncRNAs could be a potential therapeutic target for OC.

### Exosomal ncRNAs and OC Tumor Immunoregulation

The role of the immune microenvironment in tumor progression should not be ignored, including the contributions of tumor-associated macrophages (TAMs) and T lymphocytes (64). Tumor cells can evade immune surveillance and induce immune tolerance by releasing exosomes and activating other pathways, and exosome-mediated exchange of intercellular content may serve as a bridge between cancer cells and immune cells. ncRNAs in exosomes from both tumor cells and immune cells can affect the immune response (65).

On the one hand, tumor cells transfer oncogenic ncRNAs to immune cells through exosomes, which induce the dysfunction of immunosuppressive cells, thus promoting tumor growth and metastasis and other physiological activities. For example, hypoxia increased the level of miR-940 in EOC-derived exosomes, and these miR-940-rich exosomes secreted from tumors were internalized by unpolarized macrophages, driving their transformation to an M2-like phenotype (**Figure 2**) (66, 67). In addition, miR-222-3p has been shown to have the above characteristics. It is enriched in exosomes released by EOC cells and can be transferred to macrophages. Subsequently, the overexpression of miR-222-3p in macrophages induces M2 phenotypic polarization (43).

On the other hand, ncRNA-carrying exosomes from immune cells also influence tumor progression, depending on the type of immune cells. TAM-derived exosomes transferred STAT3-targeting miRNAs (e.g., hsa-miR-29a-3p and hsa-miR-21-5p) to T cells and regulated the polarization of a subpopulation of T cells, resulting in Treg/Th17 imbalance and thereby promoting tumor progression (64). The Treg/Th17 ratio was correlated with histological grade and was an independent prognostic factor for OS in patients with EOC (64).

### Exosomal ncRNAs and OC Drug Resistance

Drug resistance is an important factor leading to poor prognosis and is also an important clinical problem (68). Exosomal ncRNAs are currently the focus of research and have been found to be associated with drug resistance (69).

For example, it has been shown that OC cells can release large quantities of exosomes containing miR-1246, which are absorbed by tumorigenic cells in the tumor microenvironment, ultimately leading to paclitaxel chemotherapy resistance by inhibiting CAV1 (**Figure 2**) (32). In addition to exosomal miR-1246, exosomal miR-223 has also been shown to be associated with drug resistance (70). Specifically, EOC hypoxia upregulated the level of miR-223 in TAMS-derived exosomes, and these miR-223-rich exosomes were subsequently internalized by EOC cells, triggering chemotherapeutic resistance in EOC cells *via* the miR-223/PTEN-PI3K/AKT axis (70). In another study, it showed that the enrichment of miR-21-3p, miR-21-5p, and miR-891-5p in exosomes contributed to the drug resistance of OC to carboplatin (71). In contrast, the exosomal miR-30-5p derived from DDP-resistant OC cells increased apoptosis rate by targeting SOX9, and then, increased drug sensitivity of SKOV3/DDP cells (72).

More importantly, exosomes from stromal cells, such as CAFs or TAMs, transferred miR-21 to adjacent OC cells and increased the chemotherapy resistance of OC to paclitaxel by downregulating APAF1, the direct target of miR-21 (33). In addition, exosomal miR-146a from human umbilical cord mesenchymal stem cells increased OC cell growth and chemotherapy resistance, which was mediated by the PI3K/Akt signaling pathway *via* LAMC2 (73).

Luo et al. found that exosomal circFoxp1 was significantly elevated in EOC patients, especially in DDP-resistant EOC patients. Overexpression of circFoxp1 can promote cell proliferation and produce DDP resistance, while downregulation of circFoxp1 can inhibit cell proliferation and enhance sensitivity to DDP *in vitro* and *in vivo*. circFoxp1 positively regulates the expression of CEBPG and FMNL3 through miR-22 and miR-150-3p (74).

These findings provide new approaches for the treatment of OC, especially in terms of chemotherapy resistance. Exosomal ncRNAs can be targeted in future clinical trials as promising drugs to enhance the chemotherapy sensitivity of OC, independently or complementarily modulating chemotherapy resistance in OC therapy.

### Exosomal ncRNAs and OC Stem Cell Modification

OC is characterized by high aggressiveness, recurrence, and drug resistance, the latter two of which may be due to the presence of cancer stem cells (CSCs) (75). CSCs are a group of cells that may not be eliminated by chemotherapy but can renew themselves and differentiate into multiple lineages and can be reimplanted into tumors to cause recurrence and enhance tumors (76). Therefore, further understanding the characteristics of CSCs can contribute to potentially achieving tumor remission. In one study, it was reported that MDA-MB-231-derived exosomes increased miR-454 expression in cocultured OC cells, thus significantly increasing the number of CD44+/CD133+ cells in OC *via* the miRNA-454/PRRT2/Wnt axis (**Figure 2**) (77). It was suggested that exosomal miR-454 promoted the CSC characteristics of OC cells. However, there are very few stem cells studies on OC to date, which provides an effective direction for our future studies on exosomal ncRNAs in OC.

## Exosomal ncRNAs as Biomarkers for OC

### Exosomal ncRNAs as Diagnostic Biomarkers

Because OC is often diagnosed at an advanced stage, its survival rate is extremely low (8). Early detection and subtype determination before surgery are critical for clinicians to design effective treatment strategies for each patient, which is the goal of precision medicine (78). Therefore, it is urgent to study new biomarkers to improve the early detection rate. A number of studies have highlighted specific miRNA patterns in the exosomes of ovarian cancer patients, and all these findings suggest that exosomal miRNAs have diagnostic value (78–81).

For example, serum exosome miR-34a levels were significantly elevated in patients with early OC compared with patients with advanced OC. Patients with lymph node metastasis had significantly lower levels than patients without lymph node metastasis. Moreover, the levels of the relapse group were significantly lower than those of the non-relapse group. Therefore, serum exosomal miR-34a may be a potential biomarker to improve the diagnostic efficiency of OC (82).

Recently, Yokoi et al. combined 8 circulating serum miRNAs (miR-200a-3p, miR-766-3p, miR-26a-5p, miR-142-3p, let-7d-5p, miR-328-3p, miR-130b-3p and miR-374a-5p) into a new prediction model. The model showed high sensitivity and specificity (0.92 and 0.91, respectively) to distinguish patients with EOC and healthy controls (78). Moreover, studies have shown that most of these 8 miRNAs are packaged in extracellular vesicles, including exosomes, from ovarian cancer cells (78).

In another study, 8 miRNAs (miR-21, miR-141, miR-200a, miR-200c, miR-200b, miR-203, miR-205, and miR-214) were simultaneously elevated in serum samples and paired OC primary tumors. These miRNAs were not detected in healthy controls and were significantly different in patients with benign disease compared with those with advanced ovarian cancer, suggesting that these circulating exosomal miRNAs have clinical value in the early diagnosis of ovarian cancer (79).

Regarding exosomal miR-200b, Pan et al. conducted related studies and found that it was associated with the tumor marker CA125, which is currently routinely used for EOC screening. In addition, compared with other exosomal miRNAs analyzed, exosomal miR-200b also had the highest sensitivity (64%) and specificity (86%) in distinguishing EOC in healthy women (80). In addition, exosomal miR-375 and miR-1307 were found to have the same effect, and when the expression levels of these two miRNAs were combined with the protein biomarkers CA125 and HE4, the diagnostic value of CA125 and HE4 was improved (81).

In addition to the specific miRNA patterns in exosomes, the advantages of exosomal ncRNAs themselves suggest that they can be promising biomarkers for biological diagnosis. On the one hand, exosomal ncRNAs are easy to obtain and widely exist in body fluids (34). On the other hand, exosomes can protect miRNAs from RNase, which creates conditions for the diagnosis of OC using exosomal miRNAs (83).

From these studies, we can conclude that exosomal ncRNAs can serve as novel diagnostic biomarkers for OC. At present, there are few studies on this phenomenon, and current work is still in the preclinical stage. Therefore, multicenter and large-scale clinical studies are needed in the future.

### Exosomal ncRNAs as Prognostic Biomarkers

In addition to their value in diagnosis, exosomal ncRNAs have been used to evaluate the prognosis of OC. In several retrospective studies, circulating exosomal ncRNAs have been identified as effective prognostic markers for OC. For example, circulating exosomal circFoxp1 was significantly elevated in EOC patients, especially in DDP resistant EOC patients, its expression was positively correlated with stage, primary tumor size, lymphatic metastasis, distant metastasis, residual tumor diameter and clinical response according to the International Union of Obstetrics and Gynecology, and circFoxp1 was also an independent factor in predicting survival and disease recurrence in patients with EOC (74).

In addition, in another team’s study, they found that elevated levels of miR-373, miR-200b, and miR-200c were significantly associated with reduced overall survival (OS), and overexpression of miR-200c was also associated with shorter disease-free survival. Interestingly, increased miR-200b and miR-200c levels were also significantly associated with CA125 levels (84). In a recent study by Pan et al., the prognostic value of circulating exosome miR-200b was reinforced because they reported a significant decrease in OS when exosome miR-200b was overexpressed (80).

Another study showed that decreased serum exosome miR-484 levels were significantly associated with more invasive clinical features and shorter overall and progression-free survival. Moreover, OC patients with low expression of serum exosomal miR-484 and high expression of serum CA125 had the worst clinical outcomes. Multivariate analysis confirmed that low serum exosome miR-484 levels were an independent indicator. In conclusion, serum exosomal miR-484 can be used as a reliable and noninvasive marker for predicting the prognosis of OC (85).

In addition, high levels of exosomal MALAT1 were associated with late FIGO stage, high histological grade, and lymph node metastasis, suggesting that serum exosomal MALAT1 is associated with late and metastatic EOC behavior. Furthermore, based on multivariate survival analysis and a nomogram model, we believe that serum exosomal MALAT1 can be used as a biomarker for predicting the prognosis of EOC (26).

These results suggest that exosomal circular ncRNAs are potential prognostic markers.

## Therapeutic Functions of Exosomal ncRNAs in OC

Surgical removal of the tumor and chemotherapy are the most common treatments for OC (6). Chemotherapy resistance is largely responsible for the high mortality rate of OC (8), and the current clinical treatment of OC has limitations, which requires researchers to develop new treatment methods (86). Given the important biological functions of exosomal ncRNAs in OC, strategies that specifically target exosomes or their cargo may be a promising therapeutic option in the treatment of OC. Therefore, many current studies are focused on the characterization of exosomal ncRNAs to achieve the goal of cancer treatment.

One study showed that exosomal miR-30a-5p derived from DDP-resistant ovarian cancer cells could reduce the resistance of ovarian cancer cells to DDP (87). This study confirmed that the entry of exosomal miR-30a-5p into OC cells can enhance the sensitivity of OC cells to DDP *in vivo*: miR-30a-5p can target SOX9 in OC and downregulate SOX9, thus reversing DDP resistance. Therefore, exosomal miR-30a-5p may be a promising target for controlling drug resistance in OC (87). However, the application of this strategy may be limited by the current lack of effective exosome extraction techniques. The feasibility of this approach has yet to be demonstrated in clinical trials.

Due to difficulties in delivering ncRNAs, efforts have been made to explore engineered exosomal miRNA replacement therapies (88). In other words, exosomes were purified from omental fibroblasts of OC patients, and tumor-inhibiting miRNAs were selected and electroporated into exosomes to inhibit tumor proliferation and invasion (88). The results indicate that miRNA replacement therapy using engineered exosomes has shown a good therapeutic effect on peritoneal dissemination of OC (88). Since most OC patients undergo omentectomy, exosomes can easily be obtained from omentum fibroblasts. Engineered exosomes can be used as ncRNA delivery vectors for future molecularly targeted therapies, which may eventually lead to the development of personalized medicine for OC patients (88).

In addition to their direct involvement in the treatment of OC, ncRNAs have been shown to be involved in improving patients’ quality of life after chemotherapy (36). For example, it was found that exosomes derived from stem cells obtained from amniotic fluid contain two miRNAs, miR-146a and miR-10a, that inhibit the apoptosis of damaged ovarian granulosa cells and prevent ovarian follicular atresia in mice after chemotherapy (36). This finding reveals that miR-10a delivery may contribute to ovarian follicle preservation in female patients after chemotherapy and hints at the prospect of a cell-free therapeutic strategy rather than injecting stem cells into patients, which avoids the use of an unstable cell source and increases the safety of allogeneic donor cells.

In summary, exosomal ncRNAs have broad research prospects in the treatment of OC, but the related treatments are still in the laboratory stage and require large-scale clinical trials for confirmation (17, 35). In addition, the diagnostic potential of any biomarker depends on the type of sample (e.g., serum, urine, etc.), and the maturity of purification and detection methods will be a limiting factor (17).

## Conclusion

**Figure 3:**
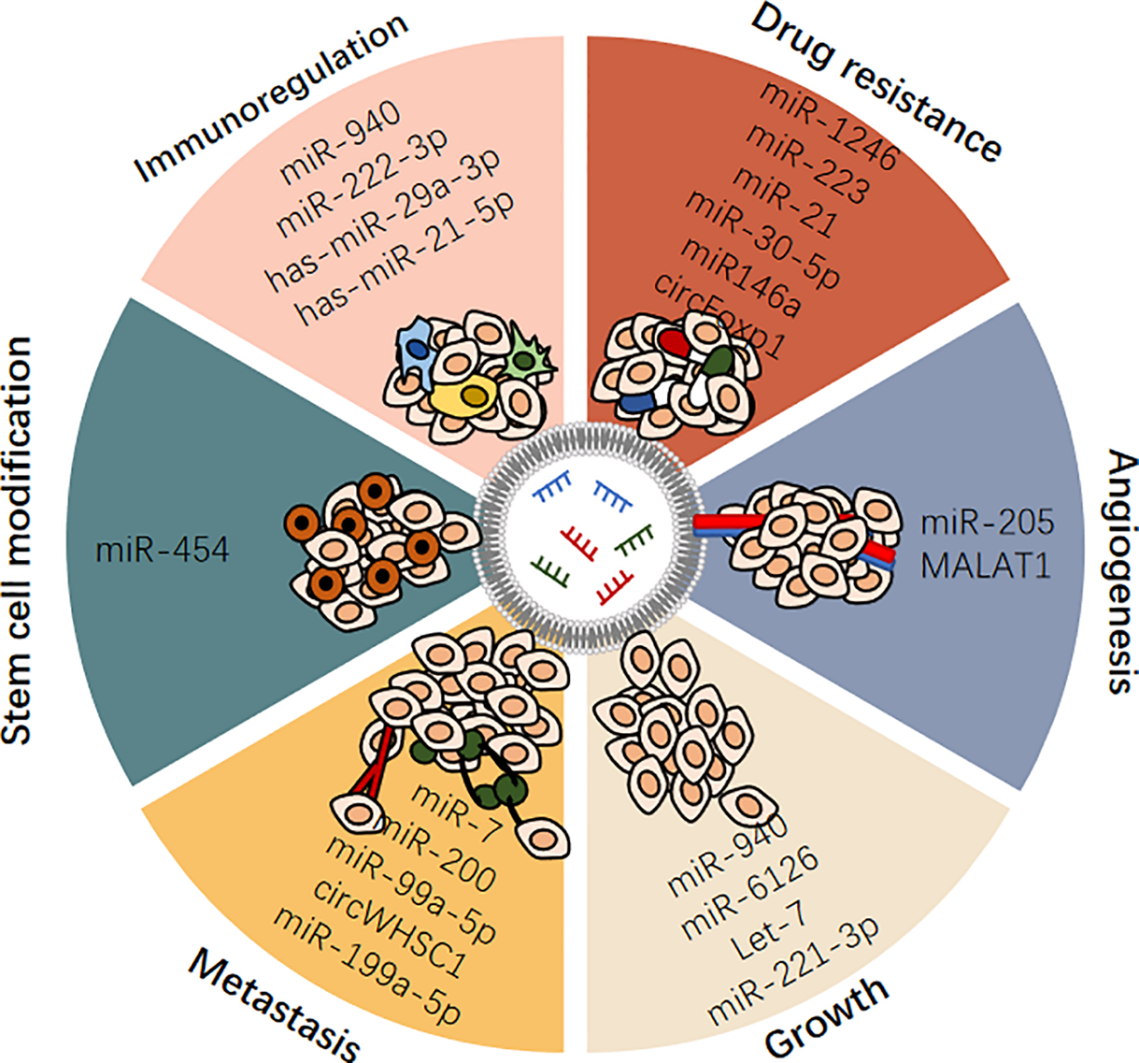
Exosomal ncRNAs play important roles in regulating ovarian cancer.

To date, OC still has the lowest survival rate of all gynecological malignancies, as it is usually diagnosed at an advanced stage. This may be due to a lack of effective tumor biomarkers. Despite advances in treatment, this disease often relapses due to resistance to chemotherapy. This is a reminder of the limitations of current OC treatments. Thus, in this paper, we reviewed the characteristics, action, and mechanism of exosomal ncRNAs, systematically summarized the role of exosomal ncRNAs in the occurrence and development of OC (**Table 1** and **Figure 3**). And we also discussed the possibility of exosomal ncRNAs as diagnostic, prognostic, and therapeutic targets, and prospected the research prospects of exosomal ncRNA in OC (**Table 1**).

**Table 1:** Summary of exosomal ncRNAs in the occurrence and development of OC.

Name	Category	Downstream Target	Function	Material Source	Year	Reference
MALAT1	lncRNA	recipient HUVECs	promote angiogenesis of human umbilical vein endothelial cells(HUVECs)	60 EOC patients recruited based on the availability of blood samples and follow-up data.	2018	(26)
circWHSC1	circRNA	E-cadherin	increased cell proliferation, migration and invasion, and inhibited cell apoptosis, provides novel ideas for new diagnostic and therapeutic strategies for clinical cancer therapy.	Seventy-nine epithelial ovarian carcinoma samples and 13 normal ovary samples	2019	(48)
circFoxp1	circRNA	miR-22 and miR-150-3p/CEBPG and FMNL3	promoting cell proliferation and produce DDP resistance, a potential biomarker and potential therapeutic target for EOC.	serum samples from 112 patients with EOC and 82 healthy people at the time of diagnosis before starting chemotherapy	2020	(72)
miR-6126	miRNA		inhibits cancer cell proliferation and tumor growth, a potential therapeutic target	ovarian tumors (n=19) and normal ovarian surface epithelium (n=6)	2016	(28)
miR-200	miRNA		associated with tumor metastasis by inhibiting epithelial-mesenchymal transformation.	the human ovarian cancer cell lines OVCAR-3 and SKOV-3	2014	(29)
Let-7	miRNA		repressing cell proliferation	OVCAR-3 and SKOV-3 (with more invasive capacity than OVCAR-3)	2014	(29)
miR-205	miRNA	PTEN/AKT	promotes metastasis by inducing angiogenesis, a potential therapeutic target	68 paraffin-embedded tissue samples (including 40 OC tissue specimens, 20 normal ovarian tissue specimens and 8 metastatic tissues) were collected from patients with OC	2019	(30)
miR-1246	miRNA	Cav1	confers paclitaxel resistance in OC cells, provide a new mechanistic therapeutic approach to overcome chemoresistance and tumor progression	OC tissue samples (n = 15) and normal ovarian surface epithelium specimens (n = 7) were obtained from patients	2018	(31)
miR21	miRNA	APAF1	Confers paclitaxel resistance, an alternative modality in the treatment of metastatic and recurrent ovarian cancer	Ovarian tissue samples were obtained from the ovarian cancer repository of the Department of Gynecologic Oncology; Normal omental tissue samples were obtained from patients with benign gynaecologic diseases	2016	(32)
miR-222-3p	miRNA		induces polarization of tumor-associated macrophages, a potential biomarker of EOC	Blood samples from 6 healthy controls and 6 pre-therapy EOC patients	2016	(41)
miR-940	miRNA		suppresses OC cell proliferation and colony formation and induces G0/G1 cell cycle arrest, maintain their invasiveness and tumorigenic phenotype.	HeyA8, SKOV3IP1, A2780-PAR, and A2780-CP20 and the non-transformed human ovarian surface epithelial cell line HIO-180	2017	(44)
miR-221-3p	miRNA	CDKN1B	participating in G1/S transition of EOC cells and playing a role in regulating EOC progression, may providing novel diagnostic biomarkers and therapeutic targets for EOC	SKOV3 and ID8 cell lines	2020	(45)
miR-99a-5p	miRNA	fibronectin and vitronectin	promoted ovarian cancer invasion and exhibited increased expression levels of fibronectin and vitronectin, a potential target for inhibiting OC progression	HPMCs were isolated from the omentum of patients	2018	(49)
miR-199a-5p	miRNA	Wnt/β-catenin	playing a negative regulatory role in tumor metastasis		2020	(53)
miR-7	miRNA		inhibit metastasis, a promising therapeutic target	human EOC cell lines SKOV3, HO-8910PM and monocyte cell line THP-1	2017	(55)
miR-6780b-5p	miRNA		promoting EMT and ultimately promoting OC metastasis	the human ovarian cancer cell lines A2780, SKOV3, CAOV3, and ES2	2021	(57)
miR-29a-3p & miR-21-5p	miRNA		induce imbalance of Tregs/Th17 cells, paving the way for the development of novel treatments for EOC	PB was obtained from 150 patients and 20 healthy donors, and ascites came from 27 EOC patients with ascites. EOC tissues were from 124 EOC patients, 15 of whom had peritoneal tissue collected at the same time. Benign tumor tissues were from 26 benign ovarian tumors patients, 6 of whom had peritoneal tissue collected at the same time.	2018	(62)
miR-940	miRNA		miR−940−induced M2 macrophages promote EOC cell proliferation and migration.	peritoneal fluids from three patients with benign ovarian diseases and ascites from three pre-therapy EOC patients	2017	(64)
miR-223	miRNA	PTEN-PI3K/AKT	elicit a chemoresistant phenotype	serous ovarian cancer samples from patients with FIGO stage IIIC or IV (n= 62)	2019	(68)
miR-21-3p	miRNA		contributing to the drug resistance of OC to carboplatin	TOV-112D, HEY, OVCA-429, SKOV-3, CAOV-3, OVCA-420, A2780, OV90 and OVCAR-3	2020	(69)
miR-21-5p	miRNA					
miR-891-5p	miRNA					
miR-30-5p	miRNA	SOX9	increasing apoptosis rate, and increasing drug sensitivity of SKOV3/DDP cells	293T and human OC cell lines (Caov3 and SKOV3)	2020	(70)
miR-146a	miRNA	LAMC2/PI3K/Akt	increasing OC cell growth and chemotherapy resistance	hUCMSCs (Sc2020011402)	2020	(71)
microRNA-454	miRNA	PRRT2/Wnt	promoting cancer stem cells (CSC) stemness *in vitro* and OC cell growth *in vivo*	SKOV3 and CoC1 cells	2020	(75)
miR-34a	miRNA		a potential biomarker to improve the diagnostic efficiency of OC, a potential biomarker of OC.	blood samples were collected from 58 epithelial OC patients directly	2020	(80)

A growing body of evidence suggests that ncRNAs, including miRNAs, lncRNAs, and circRNAs, play an indispensable role in the initiation and progression of OC. Exosomes are a novel means of intercellular communication and are rich in ncRNAs. Exosomal ncRNAs are selectively packaged, secreted, and transported to cells involved in intercellular communications in the tumor microenvironment, and they regulate many characteristics of OC. Exosomes are widely present in various fluids in the human body and stable *in vitro* because their contents are significantly protected by the lipid bilayer of the exosome membrane. These excellent characteristics make exosomes and exosomal ncRNAs ideal biomarker candidates for OC and provide new insights into the occurrence and potential therapeutic targets of OC. In addition, the delivery of tumor suppressor ncRNAs to target OC cells using engineered exosomes is a promising therapeutic strategy. These findings hold great promise for clinical applications.

Although promising, there is still a long way to go before exosomal ncRNA can be used in clinical practice, and several problems remain to be addressed. First, given the limited amount of genetic material present in biological liquid exosomes, optimizing the production, standardization and quantification of exosomal ncRNAs remains urgent. Second, ncRNAs play a complex role in a variety of physiological processes that maintain homeostasis, and systematic administration of high doses of ncRNAs may lead to serious side effects. Therefore, technology for the efficient and safe loading of ncRNAs into exosomes needs to be developed. In addition, we need to learn more about exosome biogenesis and design an optimal system to produce more exosomes to meet therapeutic needs. Last but not least, targeting strategies for exosomes need further research to achieve efficient delivery and avoid side effects.

In summary, with a better understanding of exosomal ncRNA expression patterns and pathological effects, exosomal ncRNAs are being recruited as promising biomarkers with high potential for diagnosing and treating OC. With an increasing understanding of these unknowns, we believe that exosomal ncRNAs will become more valuable in the diagnosis and treatment of OC in the near future.

## Author Contributions

YZ, Y-JW, Yi-FZ, and H-WL searched the literature. Yin-FZ provided inspiration and guidance for writing. YZ wrote the manuscript and prepared all the figures and tables. All authors contributed to the article and approved the submitted version.

## Funding

This work was supported by the National Natural Science Foundation of China (22006084), the Qingdao Applied Basic Research Project (19-6-2-49-cg), and Hubei Key Laboratory of Environmental and Health Effects of Persistent Toxic Substances (PTS2019-05).

## Conflict of Interest

The authors declare that the research was conducted in the absence of any commercial or financial relationships that could be construed as a potential conflict of interest.

## Publisher’s Note

All claims expressed in this article are solely those of the authors and do not necessarily represent those of their affiliated organizations, or those of the publisher, the editors and the reviewers. Any product that may be evaluated in this article, or claim that may be made by its manufacturer, is not guaranteed or endorsed by the publisher.
